# Dual Genotype *Orientia tsutsugamushi* Infection in Patient with Rash and Eschar, Vietnam, 2016

**DOI:** 10.3201/eid2408.171622

**Published:** 2018-08

**Authors:** Nhiem Le-Viet, Duc-Tuan Phan, Nho Le-Viet, Sinh Trinh, Muoi To, Didier Raoult, Philippe Parola

**Affiliations:** Quang Nam Central General Hospital, Quang Nam, Vietnam (Nhiem Le-Viet, Nho Le-Viet);; Aix-Marseille University, IRD, AP-HM, SSA, VITROME, IHU- Méditerranée Infection, Marseille, France (Nhiem Le-Viet, D. Raoult, P. Parola);; Quang Nam Northern Mountainous Region General Hospital, Quang Nam (D.-T. Phan, S. Trinh, M. To);; Danang University, Danang, Vietnam (Nho Le-Viet)

**Keywords:** scrub typhus, dual genotype infection, *Orientia tsutsugamushi*, eschar, rash, Vietnam, Kawasaki, Karp, vector-borne infections, bacteria, zoonoses

## Abstract

We report a dual genotype *Orientia tsutsugamushi* infection in Vietnam in 2016. The patient had fever, rash, and an eschar. The Kawasaki genotype was identified in the eschar specimen and Karp genotype in the whole blood specimen. The genotype co-infection rate for scrub typhus is unknown and should be further evaluated.

Scrub typhus is an acute febrile zoonosis caused by *Orientia tsutsugamushi* that is transmitted by larval trombiculid mites in rural areas (*1*). Scrub typhus is a major public health issue in the Asia-Pacific region and might also be present in Africa (*2*) and South America (*3*). Clinical manifestations can vary from mild symptoms to fatal disease in the absence of appropriate antimicrobial drugs (*4*). Many antigenic variants of *O*. *tsutsugamushi* exist, including Gilliam, Kato, Karp, Kawasaki, and Kuroki. This antigenic variation depends largely on the immune-dominant 56-kDa type-specific antigen (TSA) located on the surface of the bacteria membrane. Genotypes of *O. tsutsugamushi* are based on the 56-kDa TSA gene, which are commonly used to identify the diverse strains present in endemic countries (*4*–*6*). We report a case of a patient infected with 2 distinct genotypes of *O. tsutsugamushi*.

## The Study

**Figure 1:**
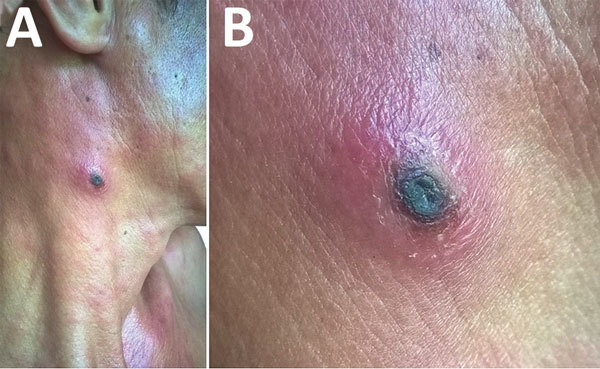
Eschar on right anterior neck of patient with dual genotype *Orientia tsutsugamushi* infection, Vietnam. A) Eschar location; B) enlarged view.

**Figure 2:**
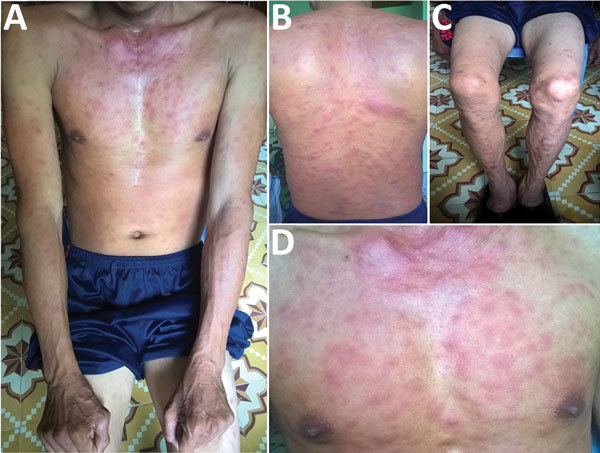
Rash on patient with dual-genotype *Orientia tsutsugamushi* infection, Vietnam. A) Trunk and arms; B) back; C) legs; D) chest.

A 35-year-old man, who often harvested agarwood in the forests of central Vietnam, had a fever and headache during a harvesting trip. On the seventh day of illness, he sought treatment at Quang Nam Northern Mountainous Region General Hospital (Quang Nam, Vietnam). He had continuous high fever, rigor, increasing headache, and muscle pain but no nausea, vomiting, or abdominal pain. A rash developed 1 day after the fever. During his time working in the forest, the man took 3 pills of acetaminophen per day to treat his symptoms, but this medicine did not improve his condition. On examination, the patient was highly febrile (39°C–40°C) and had an oval-shaped, painless eschar measuring 8 × 10 mm on his right anterior neck (Figure 1). He also had a rash covering his whole body (Figure 2). Swollen lymph nodes were observed along the mid-jugular chain of the right neck and right armpit; the largest lymph node was 10 mm in diameter, mobile, and painless. Results of routine laboratory tests for serum aspartate aminotransferase, alanine aminotransferase, and creatinine and complete blood cell counts were within reference ranges. Oral doxycycline (100 mg), prescribed immediately after scrub typhus was suspected, was administered twice a day starting on the first day of hospitalization. Defervescence occurred 24 hours after the first dose of doxycycline, and the man completely recovered.

On the day of admission, we collected a sample of the eschar by rotating a swab vigorously at the eschar base after the crust was removed, as previously described (*7*). We also took 500 µL of whole blood and 500 µL of acute-phase plasma and stored these specimens at −20°C. Specimens were transported to Marseille, France, for molecular biology and serologic testing.

We isolated DNA from the eschar and whole blood specimens and performed a real-time quantitative PCR (qPCR) specific for the periplasmic serine protease gene of *O. tsutsugamushi*; qPCR results showed these specimens were positive for the protease gene (*6*). Then, we subjected the DNA to a conventional PCR targeting the *O. tsutsugamushi* 56-kDa TSA gene using appropriate negative controls and sequenced the PCR products. We analyzed the partial 56-kDa TSA gene sequences from our patient with ABI PRISM DNA Sequencing Analysis software version 3.0 (Applied Biosystems, Foster City, CA, USA) and compared them with those available in the GenBank database using the BLAST algorithm (http://blast.ncbi.nlm.nih.gov/Blast.cgi). The DNA sequence obtained from the eschar specimen (GenBank accession no. MF769529) was closely related to the Kawasaki genotype, showing 97.2% identity to the reference strain TPC0707a (GenBank accession no. GQ332758) (*8*). The eschar specimen sequence was even more similar to a Kawasaki isolate previously detected in a patient in Quang Nam Province, Vietnam (GenBank accession no. KU871388, 98.8% identity) (*6*). The DNA sequence obtained from the whole blood specimen (GenBank accession no. MF769530) demonstrated 98.8% identity to that of a Karp isolate in Cambodia (GenBank accession no. HQ718422) (*4*). The whole blood specimen sequence was also highly similar (98.4% identity) to the sequences of 2 Karp-related isolates previously detected in patients in Quang Nam Province (GenBank accession nos. KU871384 and KU871378) (*6*). We tested the acute-phase plasma sample collected from the patient on the seventh day of illness using a micro-immunofluorescence assay (IFA) that included whole-cell antigens of *O*. *tsutsugamushi* serotypes Karp, Kato, and Gilliam. However, results were negative for specific antibodies to these antigens.

We tested for dengue virus, *Plasmodium* spp., and *Leptospira* spp. by qPCR using the whole blood specimens; negative results led to the exclusion of these pathogens as the causative agent of the fever. We also tested for other rickettsiae (*Rickettsia typhi*, *R. felis*, *R. conorii* and other spotted fever group rickettsiae, and *Coxiella burnetii*) using specific qPCRs and IFAs and excluded these pathogens as well.

Because this patient’s signs and symptoms included eschar and rash, his clinical presentation was typical for scrub typhus. A rash is observed in ≈50% of patients infected with scrub typhus (*9*), the percentage varying depending on the *O. tsutsugamushi* genotype (*6*). Eschar is associated with many rickettsial diseases and is present in 7%–80% of scrub typhus patients (*10*). Eschar is associated with severe renal, hematologic, respiratory, and circulatory manifestations; long hospital stay; and high mortality rate (*11*). The eschar is the preferred sampling site for *O. tsutsugamushi* detection and isolation. Eschar swabs are preferred over eschar biopsy and blood samples because swabbing eschars is noninvasive, easy, and painless and PCRs of DNA from eschar swab samples are highly sensitive and specific (*6*,*12*).

In 2014, *O. tsutsugamushi* genotype co-infections were reported in rodents and wild chiggers, as well as in naturally infected and laboratory-reared mites, in Thailand; the Karp genotype was detected in all mites examined, and Gilliam and UT302 were the co-infecting genotypes (*13*). Sonthayanon et al. tested whether scrub typhus patients were simultaneously infected with multiple *O. tsutsugamushi* genotypes using multilocus sequence typing (*14*); however, the specific genotypes could not be identified in their study.

## Conclusions

We demonstrated the coexistence of 2 different *O. tsutsugamushi* genotypes in this patient. The negative *O. tsutsugamushi* IFA results could have been a result of early stage plasma collection, on the seventh day of illness. For efficient serologic diagnosis, convalescent-phase plasma or serum is needed. Unfortunately, we could not obtain this type of sample from this patient because he returned to work in the forest after 5 days of treatment. Because serologic results can be negative for over a week after scrub typhus disease onset, we stress use of molecular assays rather than serologic tests for diagnosis.

We used appropriate controls and obtained sequences that had not been obtained before in our laboratory, making contamination a highly unlikely explanation for our results. However, in vitro isolation would have been stronger evidence for a dual *O. tsutsugamushi* infection.

Because this patient often worked in the forest, his dual infection could have been caused by bites from several mites, but bites from a single mite infected with multiple genotypes could have also happened (*13*,*14*). Although detecting both genotypes in the eschar sample would have been expected if he was bitten by a mite infected with multiple genotypes, 1 genotype might have predominated. Furthermore, a person bitten by multiple mites might have multiple eschars, but eschar formation varies, depending on the *O. tsutsugamushi* genotype (*15*). Wearing adequate personal protective gear when in wooded areas can help minimize exposure to mites and prevent scrub typhus. However, when a patient like such as the one we describe seeks treatment, clinicians should administer doxycycline immediately.
